# Sagittal and Vertical Changes of the Maxilla after Surgically Assisted Rapid Palatal Expansion: A Systematic Review and Meta-Analysis

**DOI:** 10.3390/jcm12103488

**Published:** 2023-05-16

**Authors:** Jia-Hong Lin, Steven Wang, Usama Al Abdullah, Anh D. Le, Chun-Hsi Chung, Chenshuang Li

**Affiliations:** 1 Department of Orthodontics, School of Dental Medicine, University of Pennsylvania, Philadelphia, PA 19104, USA; linjiah@upenn.edu (J.-H.L.); chunc@upenn.edu (C.-H.C.); 2 Department of Oral and Maxillofacial Surgery/Pharmacology, School of Dental Medicine, University of Pennsylvania, Philadelphia, PA 19104, USA; steven.wang@pennmedicine.upenn.edu (S.W.); anhle@dental.upenn.edu (A.D.L.); 3 School of Dental Medicine, University of Pennsylvania, Philadelphia, PA 19104, USA; usamasalabdullah@gmail.com

**Keywords:** SARPE, SARME, cephalometric, maxilla, sagittal, vertical

## Abstract

Surgically assisted rapid palatal expansion (SARPE) is often performed to correct the transverse deficiency in the maxilla for skeletally mature patients. However, there is little consensus on the sagittal and vertical displacement of the maxilla after SARPE. This systematic review aims to analyze the position changes of the maxilla in the sagittal and vertical dimensions after the completion of SARPE. Registered with PROSPERO (registration number: CRD42022312103), this study complied with the 2020 PRISMA guideline and was conducted on 21 January 2023. Original studies were screened from MEDLINE (PubMed), Elsevier (SCOPUS), and Cochrane, and supplemented by hand-searching. Cephalometric changes of skeletal vertical and sagittal measurements were the focus. A fixed-effects model was applied in R for meta-analysis. After applying inclusion and exclusion criteria, seven articles were included in the final review. Four studies had a high risk of bias, and the other three had a medium risk of bias. Meta-analysis revealed that the SNA angle increased by 0.50° ± 0.08° (95% confidence interval, 0.33° to 0.66°), and the SN–PP angle increased by 0.60° ± 0.09° (95% confidence interval, 0.41° to 0.79°) after SARPE. In summary, the maxilla displayed statistically significant forward and clockwise downward movement after SARPE. However, the amounts were small and might not be clinically significant. Due to the high risk of bias of included studies, our results must be taken cautiously. Future studies are needed to discern the effects of direction and angulation of the osteotomies of SARPE on the displacement of the maxilla.

## 1. Introduction

The maxillary expansion has been popular among orthodontists since the 1960s to correct crossbite in the posterior dentition [1]. However, as the resistance from the maxillary buttress increases with age, it becomes increasingly difficult to achieve skeletal expansion without detrimental effects on the teeth and periodontium when patients become older [2]. To facilitate skeletal expansion in mature patients, several treatment modalities designed to decrease bony resistance have been proposed [3,4]. Among them, surgically assisted rapid palatal expansion (SARPE) can achieve maxillary expansion through a pre-expansion LeFort 1 osteotomy without down fracture, combined with a midpalatal cut [5,6]. Then, an expander can be activated to distract the hemi-maxillae successfully. With a low relapse rate of 5–25%, SARPE is now considered a reliable method for maxillary expansion in skeletally mature patients [7].

The direction in which the maxilla is displaced after SARPE has been a topic of interest for orthodontists and surgeons [8]. However, there is little information in the literature regarding the sagittal and vertical movement of the maxilla after SARPE. A lateral cephalogram study by Chung et al. [9] reported anterior displacement of the maxilla immediately after SARPE. In addition, a downward movement of the maxilla after SARPE and orthodontic treatment was observed in a cone-beam computer tomography (CBCT) study by Xi et al. [10]. It was suggested that these movements might affect the craniofacial skeletal sagittal and vertical patterns, and the initial treatment plan [10]. On the other hand, Iodice et al. reported no skeletal sagittal or vertical variation after SARPE [11]. There is little consensus regarding the direction and amount of maxillary displacement after SARPE, and its possible effects on esthetics and function. Therefore, this systematic review and meta-analysis was conducted to analyze the changes of the maxilla in the sagittal and vertical dimensions after SARPE. We hope the results will provide clinical guidance on the outcome prediction of SARPE.

## 2. Materials and Methods

Following the 2020 Preferred Reporting Items for Systematic Reviews and Meta-Analyses (PRISMA) guideline [12], this systematic review and meta-analysis was approved by PROSPERO (registration number CRD42022312103) on 23 March 2022. Original articles were obtained from accessible electronic databases, including MEDLINE (PubMed), Elsevier (SCOPUS), and Cochrane, on 20 May 2022, and updated on 21 January 2023.

### 2.1. Study Design and Selection Criteria

A systematic review of the published studies was conducted. Conforming to PICO (population, intervention, control, and outcomes) rules (Table 1), the inclusion criteria were (a) primary study on patients with narrow maxilla (RCTs and non-RCTs, and case-series with subjects of at least 10); (b) patients who underwent SARPE; and (c) research measuring the changes of maxillary cephalometric landmarks before and after SARPE using radiographs. In addition, articles were excluded if they focused on (a) more than two-piece SARPE; (b) craniofacial anomaly; (c) additional procedures on the maxilla other than SARPE; (d) initiation of orthodontic treatment before post-surgery radiograph; and (e) nonhuman studies.

### 2.2. Search Strategy

The search was performed using the following keywords in the three electronic databases as listed above: (*surgically assisted rapid maxillary expansion OR surgically assisted rapid palatal expansion OR transpalatal distraction osteogenesis) AND (sagittal OR vertical OR asymmetry OR complications*). In addition, a manual search was conducted for articles cited by the included research. We did not define any limits on the publication date or language.

### 2.3. Data Extraction and Analysis

To ensure the reliability and completeness of the literature search results, two authors (J.-H.L. and U.A.A.) conducted the thorough literature gathering and screening process independently. Once duplicates from various databases were eliminated, the abstracts were reviewed individually to determine whether they met the inclusion and exclusion criteria. A third author (C.L.) was consulted for further deliberation if there were any discrepancies. Subsequently, the full texts were reassessed with respect to the predetermined inclusion and exclusion criteria to ensure their pertinence and applicability. In addition, data such as the expander design, expansion protocol, and timing of records were extracted. The cephalometric measurement values on the maxillary position were also extracted as follows:

Sagittal measurements:

- SNA—the angle from sella to nasion to A point.
- Nvertical to A—the distance from a vertical line passing nasion to A point.
- NA–FH—the angle between nasion–A point and Frankfurt horizontal line.
- Cf–A—the distance from the facial center to A point.
- S–A—the distance from sella to A point.
- Co–A—the distance from condylion to A point.

Vertical measurements:

- SN–PP—the angle between the sella-nasion and the palatal plane.
- FH–PP—the angle between the Frankfurt horizontal line and the palatal plane.
- N–ANS—the distance from nasion to the anterior nasal spine.
- FH–ANS—the distance from the Frankfurt horizontal line to the anterior nasal spine.
- ANS drop—the displacement of the anterior nasal spine on the vertical axis based on the cranial base superimposition.
- FH–PNS—the distance from Frankfurt horizontal line to the posterior nasal spine.
- PNS drop—the displacement of the posterior nasal spine on the vertical axis based on the cranial base superimposition.
- N–Cf–A—the angle from nasion to the facial center to A point.
- FH–A—the distance from the Frankfurt horizontal line to A point.

### 2.4. Risk of Bias/Quality Assessment

The current study employed the same risk of bias scale utilized in a prior systematic review [13] investigating the complications of SARPE to evaluate the strength of evidence presented in each of the studies included in the analysis. The evaluation criteria comprised a range of factors that are listed in Table 2. Studies that met all the criteria were deemed to have a low risk of bias and high strength of evidence. Studies that missed one or two criteria were classified as having a medium risk of bias and medium strength of evidence. Conversely, studies that failed to meet more than two criteria were deemed to have a high risk of bias and low strength of evidence.

### 2.5. Statistical Analysis

Changes in cephalometric measurements after SARPE were the primary measurement of the treatment effect. The skeletal measurements representing the vertical or sagittal position of the maxilla were extracted. Meta-analysis was performed using R 4.2.2 (R Foundation for Statistical Computing, Vienna, Austria) with the package *metafor*. A heterogeneity test was conducted, and *p* < 0.05 was defined as statistically significant. Given their similarities in design, a single study fixed-effects meta-analysis using the inverse-variance method [19] was applied.

## 3. Results

### 3.1. Literature Searching and Study Selections

**Figure 1:**
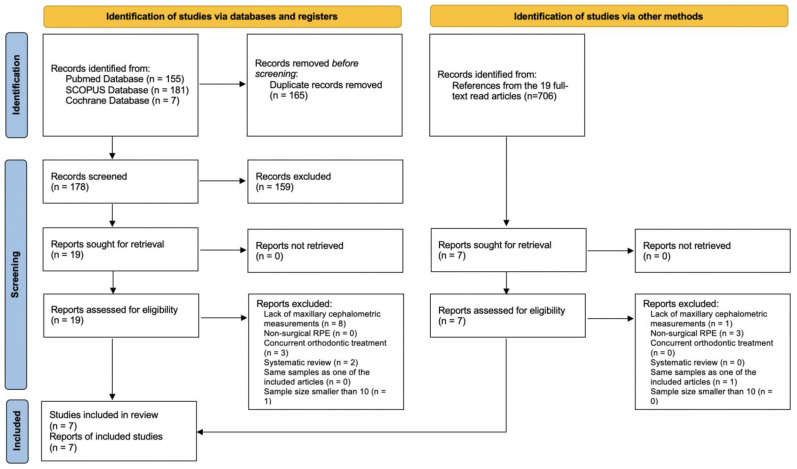
PRISMA flowchart.

Initial electronic database searching identified 343 candidate articles, which included 155 from PubMed, 181 from SCOPUS, and seven from Cochrane. Once duplicate records were removed, a title and abstract screening was conducted. Nineteen articles were identified for full-text reading. In addition, another seven articles were included after being referenced by the included articles. Ultimately, after undergoing a comprehensive assessment based on the predetermined inclusion criteria, a total of 7 articles were deemed eligible and subsequently included in the final analysis [9,11,14,15,16,17,18]. The other 19 articles were excluded due to the following reasons: nine lacked maxillary cephalometric measurements [8,13,20,21,22,23,24,25,26]; three were focused on nonsurgical RPE [27,28,29]; three included patients with orthodontic tooth movement between the two time points of obtaining radiological images [10,30,31]; two were systematic reviews [32,33]; one was reported again in a later study by the same team [34]; and one was a case series with limited sample size [35]. The PRISMA flowchart of the article inclusion and exclusion screening is shown in Figure 1.

### 3.2. Risk of Bias

The risk of bias assessment (Table 2) was conducted on all seven articles to ascertain their strength of evidence. Based on this evaluation, three of the studies were identified as having a medium risk of bias [16,17,18], while the other four studies were deemed to have a high risk of bias [9,11,14,15]. The most common sources of bias were “sample randomization” and “blind assessments”.

### 3.3. Demographics

Table 3 presents the primary characteristics of the seven articles that were included in the analysis. They included three retrospective cohort studies [14,16,17], and four retrospective case series [9,11,15,18]. The number of total patients was 136, with 126 given tooth-borne expanders and 10 given bone-borne expanders. The expansion rate noted across the selected studies varied from 0.4 mm to 1 mm per day, and activation was initiated before the end of first week after the surgical procedure. All the studies used only 2D lateral cephalograms for evaluation.

### 3.4. Maxillary Changes in the Sagittal Dimension

All the articles [9,11,14,15,16,17,18] included in the study recorded maxillary displacement in the sagittal direction after SARPE with different parameters (Table 4). Statistically significant changes were observed in the following parameters:

(a) Increase in SNA angle reported by Chung et al. [9] (0.60° ± 1.01°, *p* < 0.05), by Bretos et al. [14] (2.2°, *p* < 0.05), and by Günbay et al. [15] (1.00° ± 0.47°, *p* < 0.001);
(b) Increase in Nvertical to A distance by Chung et al. [9] (0.55 mm ± 0.78 mm, *p* < 0.05) and by Bretos et al. [14] (2.0 mm, *p* < 0.05);
(c) Increase in NA–FH angle by Chung et al. [9] (0.65° ± 0.76°, *p* < 0.05) and by Bretos et al. [14] (2.2°, *p* < 0.05);
(d) Increase in Cf–A angle by Bretos et al. [14] (1.8°, *p* < 0.05).

**Table 4:** Sagittal changes of the maxilla after SARPE. * *p* < 0.05 when comparing pre- and post- SARPE tracings. *** *p* < 0.001 when comparing pre- and post- SARPE tracings. ^&^ the changes between pre-SARPE and post-SARPE were not provided by the original study; the changes listed here were calculated based on subtracting the mean values of pre-SARPE and post-SARPE cephalometric results.

Parameters	References	Changes
SNA (°)	Chung et al. (2001) [9]	0.60 ± 1.01 *
	Bretos et al. (2007) [14]	Haas: 2.2 *; Hyrax: 1.3
	Günbay et al. (2008) [15]	1.00 ± 0.47 ***
	Kurt et al. (2010) [16]	0.18 ± 0.36
	Gungor et al. (2012) [17] ^&^	0.45
	Iodice et al. (2013) [11] ^&^	0.6
	Farfel et al. (2022) [18] ^&^	Post expansion: −0.3
		4 months after expansion: −0.7
		10 months after expansion: 0
N_vertical_ to A (mm)	Chung et al. (2001) [9]	0.55 ± 0.78 *
	Bretos et al. (2007) [14]	Haas: 2.0 *; Hyrax: 0.9
	Farfel et al. (2022) [18] ^&^	Post expansion: −0.11
		4 months after expansion: −0.23
		10 months after expansion: −0.24
NA–FH (°)	Chung et al. (2001) [9]	0.65 ± 0.76 *
	Bretos et al. (2007) [14]	Haas: 2.2 *; Hyrax: 0.8
	Iodice et al. (2013) [11] ^&^	0.3
	Farfel et al. (2022) [18] ^&^	Post expansion: −0.4
		4 months after expansion: −0.1
		10 months after expansion: −0.3
Cf–A (°)	Bretos et al. (2007) [14]	Haas: 1.5; Hyrax: 1.8 *
SN–ANS (°)	Iodice et al. (2013) [11] ^&^	0.6
S–A (mm)	Farfel et al. (2022) [18] ^&^	Post expansion: −0.6
		4 months after expansion: −0.6
		10 months after expansion: −0.3
Co–A (mm)	Farfel et al. (2022) [18] ^&^	Post expansion: 0.5
		4 months after expansion: 0.9
		10 months after expansion: 1.0

All the abovementioned cephalometric changes indicated an anterior displacement of the maxilla after SARPE.

**Figure 2:**
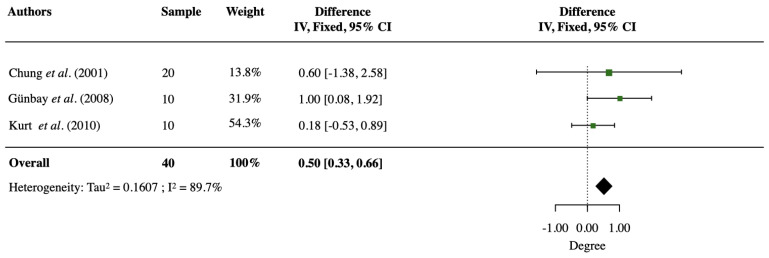
Forest plot of SNA angle (°) change after SARPE. CI stands for the confidence interval [9,15,16].

Meta-analysis could only be performed on the SNA angle change extracted from Chung et al. [9], Günbay et al. [15], and Kurt et al. [16]. As shown in Figure 2, substantial heterogeneity was found (*p* < 0.0001, *I*^2^ = 89.7%). The meta-analysis revealed that the SNA angle increased by 0.50° ± 0.08° (95% confidence level, 0.33° to 0.66°), indicating a small but statistically significant forward movement of the maxilla introduced by SARPE.

### 3.5. Maxillary Change in the Vertical Dimension

All of the included articles, except for Günbay et al. [15], reported vertical changes of the maxilla after SARPE (Table 5). Among the reported parameters, statistically significant difference was only observed in N–ANS distance by Kurt et al. [16] (1.21 mm ± 0.28 mm, *p* < 0.01), which indicated a downward movement of the anterior maxilla after SARPE.

**Figure 3:**
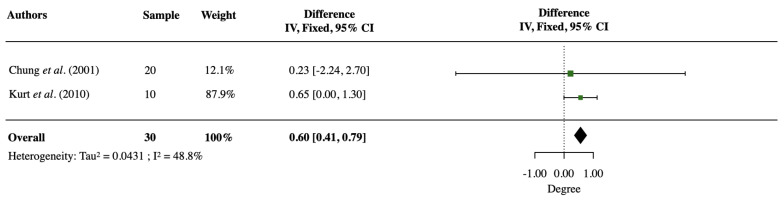
Forest plot of SN–PP angle (°) change after SARPE. CI stands for the confidence interval [9,15].

A meta-analysis was performed with two of the studies [9,16] reporting the mean and S.D. value of the SN–PP angle change. Heterogeneity across the studies was moderate (*p* = 0.126, *I^2^* = 48.8%). The meta-analysis showed an SN–PP angle increase of 0.60° ± 0.09° (95% confidence level, 0.41° to 0.79°) (Figure 3). This change indicated a small but statistically significant downward and clockwise rotation of the maxilla induced by SARPE.

## 4. Discussion

### 4.1. Expander Type

The design of the expander varied among the different studies included in our study. However, when comparing different types of tooth-borne expanders, Bretos et al. [14] demonstrated no statistically significant differences between the Haas expander and the Hyrax expander on the cephalometric change after SARPE and concluded that both expanders performed similarly.

Similar studies also compared the effects of SARPE under different expander designs. Koudstaal et al. [30] split their subjects into three subgroups according to the expander design, including Hyrax, bone-borne transpalatal distractor, and bone-borne Rotterdam palatal distractor. Under the same surgical and expansion protocol, there was no significant difference in changes in lateral cephalometric landmarks among different types of expanders [30]. Xi et al. [10] also performed a multivariate linear regression analysis with backward elimination to test the effects of the expander design (tooth-borne Hyrax vs. bone-borne transpalatal distractor) and found no significant differences.

Based on these findings, we did not differentiate cephalometric changes based on expander design in this systematic review.

### 4.2. Sagittal Change of the Maxilla

**Figure 4:**
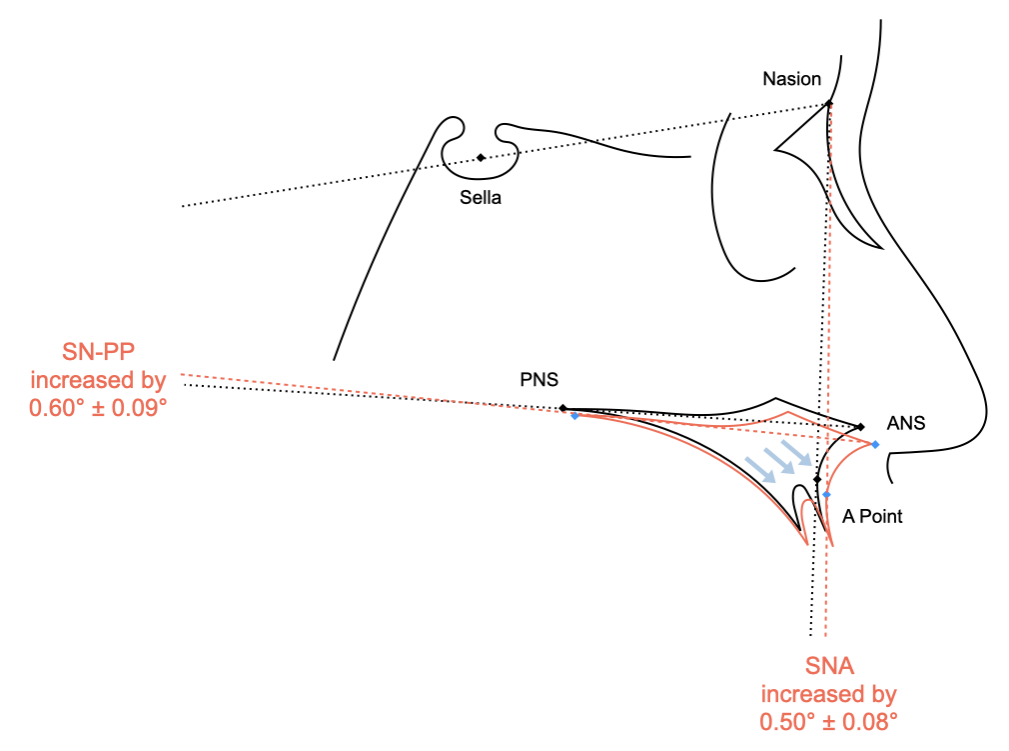
The maxilla moved downward and forward (blue arrow) after SARPE. Meta-analysis showed an increase in SN–PP angle (°) and SNA angle (°) after surgery (red line).

All statistically significant changes in the sagittal measurements included in this study pointed to an anterior displacement of the maxilla after SARPE (Table 4), including an increase in SNA angle, N_vertical_ to A distance, NA–FH angle, and Cf–A distance. However, even with some statistically significant changes, these measurements were insufficient to result in clinically significant differences. Our meta-analysis also showed that the anterior displacement of the maxilla measured by the SNA angle is limited (Figure 2 and Figure 4). Thus, clinically significant sagittal correction of malocclusion should not be expected from SARPE [9,11,14]. In other words, clinicians should not expect SARPE to be able to spontaneously correct a retrognathic maxilla. The use of reverse headgear or more complex surgeries (e.g., maxillary advancement and/or mandibular setback) may be necessary for skeletal class III correction in conjunction with SARPE [9,11,14].

### 4.3. Vertical Change of the Maxilla

Meta-analysis of the SN–PP angle pointed to a downward movement and clockwise rotation of the maxilla after SARPE (Figure 3 and Figure 4). Chamberland and Proffit [36] attributed the downward movement of the palatal process to the rotation of the hemi-maxillae and inward movement of the alveolar edges below the osteotomy sites when viewed in a posteroanterior (PA) cephalogram. Koudstaal et al. [30] explained the downward movement of the maxilla by the direction of the lateral osteotomy, which is often slanted inferiorly as it extends from the nasal aperture to the zygomatic buttress. When the hemi-maxillae were being expanded, they slid downward against the cut [30]. To prevent an inferior displacement, Betts et al. [6] proposed removing part of the zygoma and performing horizontal osteotomies to reduce interference during expansion. However, significant inferior movement of the maxilla was also observed by Kurt et al. [16], with their osteotomies performed horizontally. Thus, future studies are needed to determine the relationship between the displacement of the hemi-maxillae and the angulation of the osteotomy or obstruction by the zygoma and other surrounding hard tissues.

On the other hand, Bretos et al. [14] observed an upward, though insignificant, movement of the maxilla after SARPE. The authors stated that postsurgical bony consolidation at the osteotomy site created by a 2 mm diameter bur caused the maxilla to impact, thereby resulting in a decrease in their vertical cephalometric measurements [14].

Although not included in our study, the esthetic effects of downward movement of the maxilla were also investigated by Xi et al. [10], where 87% of their patients displayed an increase of dental show by 2.7 mm ± 1.8 mm one year after SARPE. However, given that these patients also underwent orthodontic treatment, they were unable to identify whether the increase in the dental display was due to SARPE or orthodontic treatment.

### 4.4. Limitations

Despite the widespread and extensive application of SARPE in the orthodontic and oral–maxillofacial surgical field for decades, there is a limited amount of papers focusing on the three-dimensional movement of the maxilla post-SARPE. Thus, only seven articles could be included in the current study. In addition, the absence of standard deviation in some of the included studies limited the scope of our meta-analysis. It is noteworthy that three-dimensional measurements are essential during initial diagnosis and progress evaluation for precise and optimized clinical care. We hope our current study summarized current knowledge on the sagittal and vertical impacts on the maxilla from SARPE—a surgical technique only intended to address the deficiency on the transverse dimension. We also hope the current study could stimulate worldwide collaboration to evaluate the maxilla and mandible changes in 3D after SARPE in depth, especially with the current booming in 3D imaging in the dental field.

Factors that affected our study included differences in the surgical technique (e.g., the direction of osteotomy, the release of pterygomaxillary fissure or not, etc.) and expansion protocol (activation rate and total expansion amount) among the studies, which introduced a significant amount of heterogeneity when comparing the data reported in these studies. However, since the amount required for SARPE is tailored to each individual’s transverse discrepancy, it is difficult to find multiple studies employing exactly the same protocol and total amount of expansion. Although this stands as a limitation to a systematic review, it would also be challenging to conduct a clinical study with a uniform expansion amount for all the included subjects. It is worthwhile to note that, although most of the included studies were case series and categorized as a moderate to high risk of bias, randomization is difficult to implement since SARPE is a surgical procedure that is often clearly indicated over other forms of treatment.

In the current study, we specifically excluded the studies that had their post-SARPE radiograph taken after orthodontic treatment to rule out the influences from dental movement or from orthodontic force. Most of the included studies had an average subject age above 20, with few including teenage patients. Although craniofacial growth can last up to 24 years old in some individuals [37], since the time intervals between pre- and post-SARPE radiographs of the included studies are within six months, with most of them even a few weeks apart, the influence of growth in the current evaluation, if any, is probably negligible.

Lastly, although this study did not focus on the changes in mandibular cephalometric landmarks, several of the included studies shed light on how the maxillary movement introduced by SARPE affected the rotation of the mandible. For instance, both a significant increase in the mandibular plane angle [10,15,16] and a significant decrease in SNB angle [15,16] were recorded. This downward and backward rotation of the mandible was similar to traditional nonsurgical rapid palatal expansion [10,15]. It could be attributed to the downward movement of the maxilla, tipping and extrusion of the maxillary posterior dentition, and cuspal interference [10,15]. Therefore, the effects of SARPE on mandibular position should be further explored.

## 5. Conclusions

The maxilla displayed a forward movement as well as a downward and clockwise rotation after SARPE (Figure 4). While these changes were statistically significant, the amounts of movements have a questionable amount of clinical significance. Future studies are needed to discern the effects of direction and angulation of the osteotomies on the displacement of the maxilla.

## Figures and Tables

**Table 1:** PICO questions of the current study.

Criteria	Description
Population	Patients with transverse maxillary deficiency and without craniofacial anomaly
Intervention	Two-piece SARPE
Comparisons	Pre- and post-SARPE cephalometric measurements
Outcome	Maxillary position change in the vertical and sagittal dimensions

**Table 2:** Risk of bias assessment of the 7 included articles.

	Chung et al. (2001) [9]	Bretos et al. (2007) [14]	Günbay et al. (2008) [15]	Kurt et al. (2010) [16]	Gungor et al. (2012) [17]	Iodice et al. (2013) [11]	Farfel et al. (2022) [18]
Sample randomization	No	No	No	No	No	No	No
Comparison between treatments	No	Yes	No	Yes	Yes	No	No
Blind assessments	No	No	No	No	No	No	Yes
Validation of measurements	Yes	Yes	Yes	Yes	Yes	Yes	Yes
Statistical analysis	Yes	Yes	Yes	Yes	Yes	Yes	Yes
Defined inclusion and exclusion criteria	No	No	Yes	Yes	Yes	Yes	Yes
Report of follow-up	Yes	Yes	Yes	Yes	Yes	Yes	Yes
Risk of bias	High	High	High	Medium	Medium	High	Medium

**Table 3:** Characteristics of included studies. B.B.: bone-borne maxillary expander; T.B.: tooth-borne maxillary expander; y: years; Post-Sx: post-surgery.

Author	Number	Age (y)	Method	Device	Expansion during Surgery (mm)	Expander Activation Protocol after Surgery
Chung et al. (2001) [9]	20 (6 M, 14 F)	25.6 (14.9–43.0)	Retrospective Case Series	T.B.	1–1.5	0.5 mm/day for about 2 weeks until the jackscrew was fully opened.
Bretos et al. (2007) [14]	33 (14 M, 19 F)	25 (18–40)	Retrospective Cohort Study	T.B. Haas (16)	1.6	0.4 mm/day started 4 days post-Sx until planned expansion was reached.
				T.B. Hyrax (17)		
Günbay et al. (2008) [15]	10 (6 M, 4F)	22.3	Retrospective Case Series	B.B.	-	1 mm/day started 7 days post-Sx until appropriate posterior overbite was achieved.
Kurt et al. (2010) [16]	10 (7 M, 3 F)	19.01 (16.25–25.58)	Retrospective Cohort Study	T.B.	-	0.5 mm/day until desired expansion was reached.
Gungor et al. (2012) [17]	14 (4 M, 10 F)	19.6 ± 2.73	Retrospective Cohort Study	T.B.	1	0.5 mm/day started 7 days post-Sx until the necessary amount was achieved.
Iodice et al. (2013) [11]	21 (7 M, 14 F)	25.6 ± 6.3 (20.2–30.1)	Retrospective Case Series	T.B.	1.6	0.4 mm/day started 4 days post-Sx until planned expansion was reached.
Farfel et al. (2022) [18]	28 (14 M, 14 F)	25.8 (19–39)	Retrospective Case Series	T.B.	1.6	0.4 mm/day started 4 days post-Sx until desired expansion was reached.

**Table 5:** Vertical changes of the maxilla after SARPE. ** *p* < 0.01 when comparing pre- and post- SARPE tracings. ^&^ the changes between pre-SARPE and post-SARPE were not provided by the original study; the changes listed here were calculated based on subtracting the mean values of pre-SARPE and post-SARPE cephalometric results. ^#^ standard deviation was calculated using the sample size and standard error provided by the study.

Parameters	References	Changes
SN–PP (°)	Chung et al. (2001) [9]	0.23 ± 1.26
	Kurt et al. (2010) [16]	0.65 ± 0.33
	Iodice et al. (2013) [11] ^&^	0.4
	Farfel et al. (2022) [18] ^&^	Post expansion: −0.07
		4 months after expansion: −0.07
		10 months after expansion: 0.3
FH–PP (°)	Iodice et al. (2013) [11] ^&^	1.0
N–ANS (mm)	Kurt et al. (2010) [16]	1.21 ± 0.28 **
	Gungor et al. (2012) [17] ^&^	−0.11
FH–ANS (mm)	Bretos et al. (2007) [14]	Haas: 0.3; Hyrax: 0
	Farfel et al. (2022) [18] ^&^	Post expansion: −0.1
		4 months after expansion: −0.4
		10 months after expansion: −0.1
ANS drop (mm)	Chung et al. (2001) [9]	0.45 ± 1.10
FH–PNS (mm)	Bretos et al. (2007) [14]	Haas: 0.1; Hyrax: −0.3
	Farfel et al. (2022) [18] ^#^	Post-expansion: 1.37 ± 0.10
		4 months after expansion: 1.22 ± 0.12
		10 months after expansion: 0.51 ± 0.09
PNS drop (mm)	Chung et al. (2001) [9]	0.60 ± 0.90
N–Cf–A (°)	Bretos et al. (2007) [14]	Haas: −0.9; Hyrax: −0.5
FH–A (mm)	Farfel et al. (2022) [18] ^&^	Post expansion: −0.1
		4 months after expansion: −0.7
		10 months after expansion: −0.4

## Data Availability

No new data were created or analyzed in this study. Data sharing is not applicable to this article.
